# Contribution of integrin adhesion to cytokinetic abscission and genomic integrity

**DOI:** 10.3389/fcell.2022.1048717

**Published:** 2022-12-12

**Authors:** Bhavna Rani, Deepesh K. Gupta, Staffan Johansson, Siamak A. Kamranvar

**Affiliations:** ^1^ Department of Medical Biochemistry and Microbiology (IMBIM), Biomedical Center, Uppsala University, Uppsala, Sweden; ^2^ Department of Pediatrics, Washington University School of Medicine, St. Louis, MO, United States

**Keywords:** integrin, centrosome, mitosis, abscission, genome integrity

## Abstract

Recent research shows that integrin-mediated adhesion contributes to the regulation of cell division at two key steps: the formation of the mitotic spindle at the mitotic entry and the final cytokinetic abscission at the mitotic exit. Failure in either of these processes will have a direct impact on the other in each round of the cell cycle and on the genomic integrity. This review aims to present how integrin signals are involved at these cell cycle stages under normal conditions and some safety mechanisms that may counteract the generation of aneuploid cells in cases of defective integrin signals.

## 1 Introduction

In contrast to cancer cells, normal mammalian cells depend on signals generated from integrin contacts with the extracellular matrix (ECM) for their survival and proliferation. Integrins do not have intrinsic enzyme activity but trigger several signaling pathways through ligand-induced clustering, which *via* the integrin-associated talin-kindlin-paxillin complex, brings focal adhesion kinase (FAK) together for trans-autophosphorylation at Tyr397 (Acebron I et al., 2020; Lu F et al., 2022) and the subsequent activation of Src family kinases (Calalb M B et al., 1995; Mitra S K et al., 2005). In addition, the mechanical force exerted on integrin contacts can induce signals by conformational changes in stretch-sensitive proteins (Martino F et al., 2018). The integrin adhesion-dependent FAK-Src activation is a long-known requirement for sustained induction of the PI3K-Akt and the Ras-ERK pathways needed to avoid apoptosis and to pass the G1-S checkpoint of the cell cycle (Schwartz and Assoian, 2001; Reddig and Juliano, 2005). The absence of the adhesion will efficiently stop proliferation unless the checkpoint is suppressed by viruses or mutations (Thullberg and Stromblad, 2008; Deng Z et al., 2021; Engeland K, 2022). More recently, integrin signals *via* FAK have been found to contribute to cytokinetic abscission (Reverte C G et al., 2006; Thullberg M et al., 2007; Pellinen T et al., 2008; Kamranvar S A et al., 2016), as well as to the arrangement of centrosomes into a bipolar mitotic spindle which is also essential for successful cytokinesis (Park A Y et al., 2009; Kamranvar S A et al., 2022b). Although failures at these stages will cause extensive cell death either immediately or in the following cell cycles, they will not serve as reliable protection mechanisms against unwanted proliferation (Hoevenaar et al., 2020; Vasudevan A et al., 2021). In such situations, the centrosomes become the key players for three different outcomes after the failed cytokinesis: i) the presence of two (or more) mature centrosomes can potently induce p53-dependent G1 senescence *via* the PIDDosome complex (Fava L L et al., 2017), ii) in the absence of functional p53, the presence of four (or more) centrosomes after passage through S phase will cause the formation of a multipolar spindle resulting in mitotic cell death (Rizzotto D et al., 2021), or iii) if cells with inactivated p53 cluster the centrosomes into a pseudo-bipolar spindle, viable tetraploid or near-tetraploid daughter cells will be generated; such cells tend to promote tumor formation and progression due to increased frequency of chromosomal segregation errors and aneuploidy (Fujiwara T et al., 2005; Baudoin N C et al., 2020). The current review discusses the role of integrin signals in the mitotic exit of mammalian cells, as well as the impact of their dysfunction in the context of centrosome activities and genomic integrity.

## 2 Spindle formation

An early critical step for the onset of mitosis, as well as for mitotic exit and the following cytokinesis, is the formation of a bipolar microtubule (MT) spindle by two centrosomes. The centrosome, consisting of a pair of centrioles surrounded by the pericentriolar matrix (PCM), duplicates once every cell cycle. Post duplication in S-phase, the two centrosomes remain connected through a fibrous linker of rootletin polymers and several associated proteins (Vlijm R et al., 2018), thereby acting as a single major microtubule-organizing center. During the late G2 and early mitotic (M) phases, the two centrosomes need to be separated and moved apart to serve as spindle poles. Centrosome separation (disjunction) is induced by PLK1, a key regulator of the process, through the activation of downstream kinases, among which NEK2A has the main role in the dissociation of the rootletin linker from the parental centrioles (Mardin B R et al., 2011; Hossain D et al., 2020). Once the linker is broken, the centrosome migration and positioning depend on the net balancing forces exerted by several motor proteins on MTs. The mitotic kinesin-5 (Eg5) and dynein are the two major motor proteins crucial for centrosome segregation and proper spindle bipolarity (Van Heesbeen R G et al., 2014). PLK1, together with CDK1 and NEKs 6, 7, and 9, activate Eg5 and thereby induce the translocation of the centrosomes to opposite sides of the nucleus (Bertran M T et al., 2011). Our recent findings show that integrin-mediated adhesion *via* FAK participates in the regulation of PLK1 and Eg5 and the subsequent centrosome translocation. In case of insufficient FAK signaling due to downregulated protein expression or cell detachment, cells often form a monopolar spindle and exhibit a strongly delayed mitotic exit (Kamranvar S A et al., 2022a). PLK1 activity is known to be controlled by phosphorylation at Thr210 in the activation loop by Aurora A (Gheghiani L et al., 2017; Raab M et al., 2022), and additional PLK1 modifications have been reported, which may further contribute to its spatiotemporal regulation (Caron D et al., 2016; Liu X et al., 2016; Yang X et al., 2017). Also, the regulation of Aurora A is complex, and several different activation mechanisms have been elucidated (Zhao Z S et al., 2005; Tavernier N et al., 2021). Thus, while the mechanism by which FAK promotes PLK1 activity remains to be identified, it is likely to act in parallel with or possibly upstream of Aurora A.

## 3 Cytokinesis

Cytokinesis, i.e., the division of the cytoplasm following karyokinesis, is usually considered a separate cell cycle stage and not a part of mitosis. However, the cytokinesis process is closely linked to mitosis and starts during anaphase (Echard and O’farrell, 2003). Cytokinesis can be generally divided into three stages: cleavage furrow formation and ingression, midbody assembly, and abscission. CDK1 inactivation in the late mitosis is the early sign of cytokinesis onset, leading to mitotic spindle re-organization. The anaphase spindle initiates the actomyosin ring assembly linked to the plasma membrane, whose contraction gives rise to cleavage furrow formation and ingression. Eventually, the ingressed furrow will separate the two nascent daughter cells by a thin intercellular bridge, which at its center forms a dense structure called midbody (MB). The MB serves as a signaling platform to assemble the proteins needed for severing the intercellular bridge, i.e., the abscission (Fededa and Gerlich, 2012).

In pioneering works (Thullberg M et al., 2007; Pellinen T et al., 2008), integrin-mediated adhesion was found to be required for cytokinesis completion in several mouse and human cell lines, and subsequently, it was shown that the process was halted at the abscission stage in the detached cells (Gupta and Johansson, 2013). Further work identified the integrin-dependent step to the recruitment of the ESCRT-associated proteins Alix and TSG101 to the MB (Kamranvar S A et al., 2016).

### 3.1 Cleavage furrow formation and ingression

**FIGURE 1:**
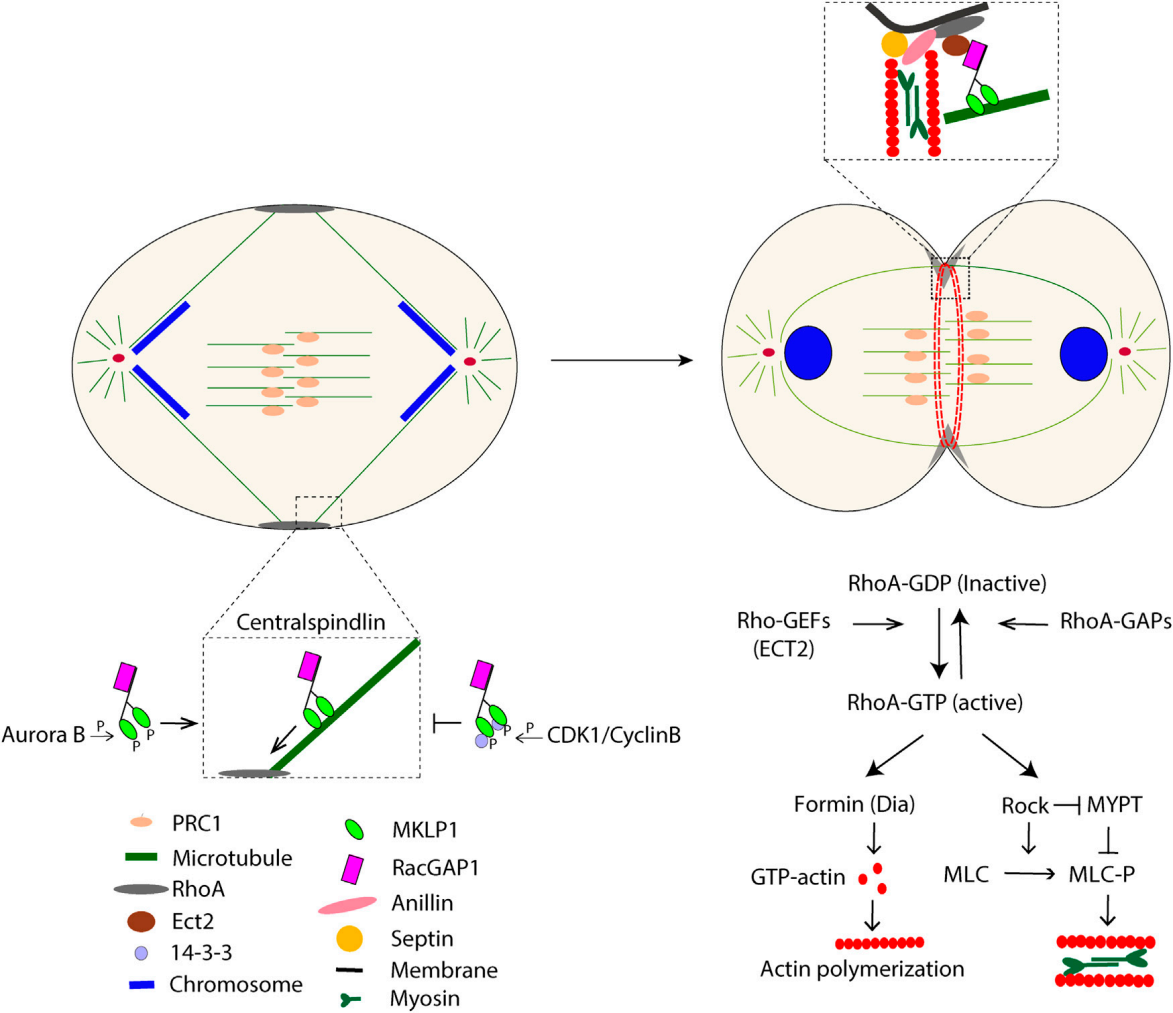
The schematic picture illustrates some major events during cytokinesis that start at anaphase.

Once the spindle assembly checkpoint has been satisfied by the completed binding of all chromatid kinetochores to MTs, the anaphase-promoting complex/cyclosome (APC/C) with its specific factor Cdc20 marks two key substrates for proteasomal degradation, i.e., securin and cyclin B (Peters J-M, 2002). During the subsequent segregation of chromosomes, a cleavage furrow ingression will be induced midway between the two spindle poles. In animal cells, the furrow formation and ingression are the synergistic outcomes of signaling and mechanical processes (Bement W M et al., 2005; Bringmann and Hyman, 2005). In the early cytokinesis, *de novo* formed MTs, together with existing MTs, assemble into the central spindle, a structure of anti-parallel MTs with overlapping plus end regions in the center of the cell between the segregating chromosomes. (Glotzer M, 2009; Uehara R et al., 2009). The central spindle is organized by MT-associated proteins, among which the roles of PRC1 and centralspindlin are well understood (Hu C-K et al., 2012b; Adriaans I E et al., 2019). PRC1 is inhibited until anaphase onset by CDK1/cyclin B and PLK1 phosphorylation, but then the declining levels of these kinases allow dimerization of PRC1. PRC1 dimers specifically recognize antiparallel MT overlaps at the central spindle, promoting MTs sliding and binding of essential central spindle-associated proteins (Mollinari C et al., 2002; Subramanian R et al., 2010). The central spindle structure and function depend on the heterotetrameric protein centralspindlin, comprised of a pair of the kinesin motor protein MKLP1 and a pair of the Rho-family GTPase RacGAP1 (Mishima M et al., 2002). Centralspindlin forms larger clusters and migrates towards the plus end of MTs. The clustering is inhibited by the binding of protein 14-3-3 to a CDK1- phosphorylated site in MKLP1, but upon the decline of CDK1/cyclin B, 14-3-3 dissociates from MKLP1 (Mishima M et al., 2004). The centralspindlin clustering is further stimulated by Aurora B-mediated phosphorylation of MKLP1 (Guse A et al., 2005). The transport of ECT2 (a Rho GEF) by centralspindlin along astral MTs determines the ingression furrow location at the cell cortex (Basant A et al., 2015; Basant and Glotzer, 2018). After GTP-loading by ECT2, Rho A will bind and activate multiple effectors that are essential for furrow ingression, including the formin Dia1 and ROCK; Dia1 induces actin polymerization, and ROCK stimulates myosin activity through phosphorylation of myosin light chain (MLC) and MLC phosphatase (MYPT) (Severson A F et al., 2002; Matsumura F, 2005). The contractile ring is connected to the plasma membrane mainly *via* the anillin-septin linker, whose formation is promoted by RhoA-GTP and citron kinase (Oegema K et al., 2000; Maddox A S et al., 2007; El-Amine N et al., 2019) (Figure 1).

### 3.2 Midbody formation

The cleavage furrow continues to ingress until it approaches a diameter of 1–2 μm, a prerequisite for the formation of the MB, whose composition gradually develops and will eventually contain hundreds of different proteins (Capalbo L et al., 2019; Addi C et al., 2020). During the membrane ingression, several components of the actomyosin ring and the central spindle rearrange their localization, contributing to the initiation of the MB assembly (Hu C-K et al., 2012a). PRC1 localizes to the MB region, and the centromere-associated proteins Aurora B and CENP-E re-localize to the flanking region of the MB (Yen T J et al., 1991; Gruneberg U et al., 2004). MKLP1 motor activity brings the centralspindlin towards the MT plus end of the intercellular bridge, where it recruits the major MB adapter protein Cep55 (Fabbro M et al., 2005; Zhao W-M et al., 2006). The recruitment of Cep55 is negatively controlled by PLK1 and occurs only after the inactivation of PLK1 during the anaphase (Lindon and Pines, 2004; Bastos and Barr., 2010), and correct timing of the PLK1 degradation is crucial for cytokinetic abscission (Bastos and Barr, 2010). We recently found that the temporal regulation of PLK1, and thus Cep55 recruitment to the MB region, depends on FAK signaling downstream of integrin adhesion. In detached cells, PLK1 is degraded faster and Cep55 accumulates prematurely, resulting in an “immature” MB that, for unknown reasons, cannot support abscission by recruiting ESCRT complex subunits (Kamranvar S A et al., 2016). Thus, the emerging daughter cells will remain connected by a thin intercellular bridge without proper adhesion signaling.

### 3.3 Abscission

**FIGURE 2:**
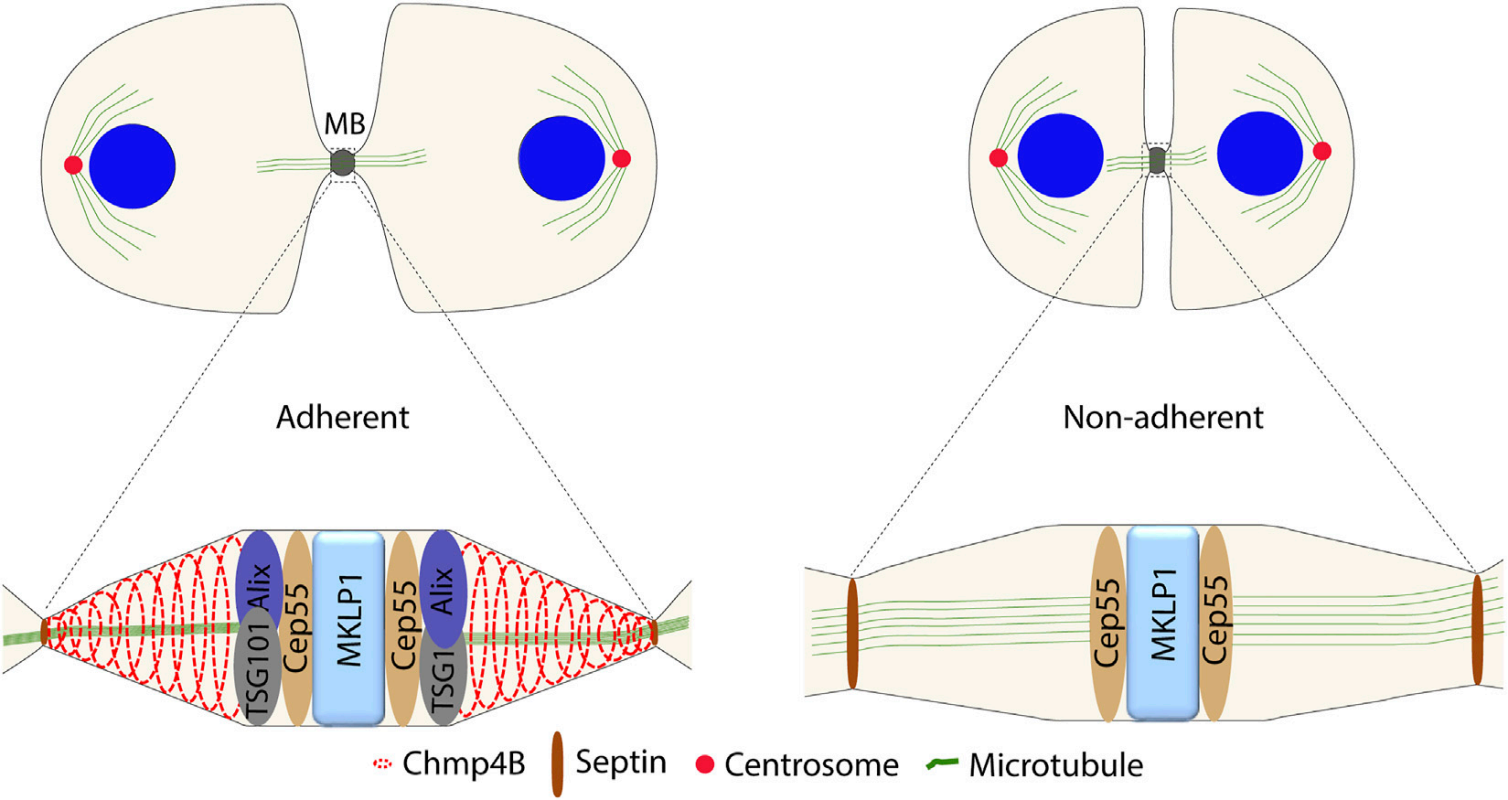
Difference in the protein composition at the MB between the adherent and non-adherent cells during abscission.

During the MB maturation, the ingression furrow narrows further to less than 1 μm. Abscission is executed by ESCRT proteins and their binding partners, which are recruited to the MB region stepwise (Mierzwa and Gerlich, 2014). Mature Cep55 recruits Chmp4B, the main component of ESCRT-III, *via* two routes with partially redundant functions. On one route, Chmp4B is recruited through ESCRT-I, ESCRT-II and Chmp6 (an ESCRT-III subunit), where Cep55 interacts with TSG101 (an ESCRT-I subunit), while in the other route *via* Cep55–Alix–Chmp4B (Christ L et al., 2016). Both TSG101 and Alix are dependent on integrin-induced FAK signaling for their Cep55-dependent recruitment to the MB (Kamranvar S A et al., 2016) (Figure 2). Alix and TSG101 cannot be recruited by Cep55 at the MB in detached cells, perhaps due to the absence of other necessary proteins or protein modifications. Due to the complex and changing composition of MBs during the cytokinesis process, finding the answer will be challenging.

To bind Chmp4B, Alix has to be activated from a closed to an open conformation induced by phosphorylation; the activating kinase remains to be identified (Sun S et al., 2016). Upon recruitment to the MB, Chmp4B will polymerize into a ring-like structure and then further polymerizes into spiral filaments of decreasing diameter that extend away from the MB. This spiral causes the narrowing of the intercellular bridge and promotes the membrane fusion process, possibly by deforming the membrane followed by depolymerization or rearrangement of the Chmp4B spiral (Guizetti J et al., 2011; Mccullough J et al., 2013; Harker-Kirschneck L et al., 2019; Harker-Kirschneck L et al., 2022). The spiral formation can be delayed or even prevented by pulling force exerted on the intercellular bridge, and abscission is promoted by tension relief through the accumulation of caveolae close to the MB (Lafaurie-Janvore J et al., 2013; Andrade V et al., 2022). At the abscission site, Alix is vital in connecting the spiral with the plasma membrane and stabilizing it by forming a link to the transmembrane proteoglycan syndecan four *via* syntenin (Addi C et al., 2020). Before the membranes can finally fuse, the dense MTs at the bridge have to be cleared away by spastin, an MT-severing enzyme targeted to the constriction region *via* binding to the ESCRT-III subunit Chmp1B (Yang D et al., 2008; Connell J W et al., 2009).

### 3.4 Cytofission

**FIGURE 3:**
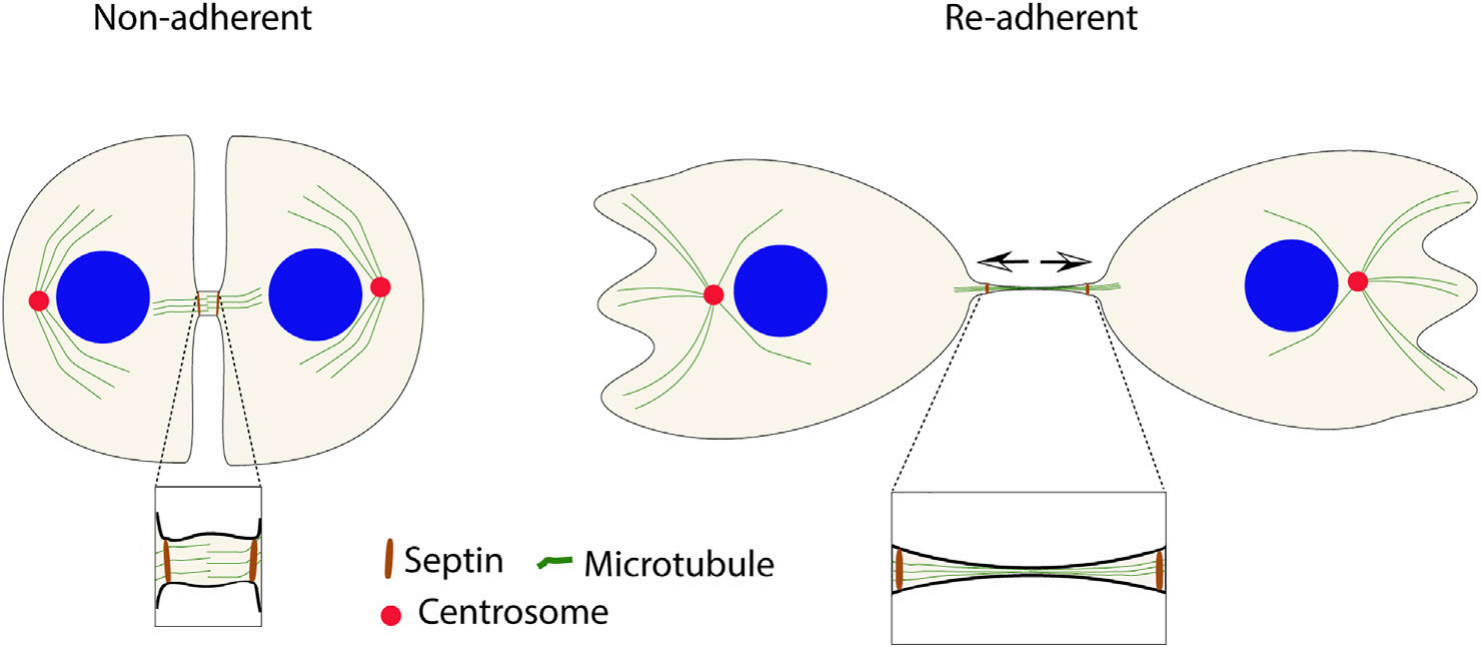
Cytofission is an alternative mechanism to fulfill the incomplete cytokinesis in non-adherent cells after re-adhesion. MB-independent fission can occur due to the stability of the furrow by septin filaments (in brown). The arrows show the direction of tension in the connecting bridge.

In addition to the highly regulated abscission process, cells can, under particular circumstances, divide by so-called cytofission independently of the MB (Gupta D K et al., 2018; Gupta D K et al., 2019). Cytofission instead depends on traction force in adherent cells which can eventually rupture the ingression bridge. Thus, the pulling force has opposite effects on cell division *via* MB-mediated abscission and cytofission. The cytofission mechanism has mainly been studied in the slime mold *Dictyostelium* (Tuxworth R I et al., 1997; Chung and Firtel, 1999; Kanada M et al., 2005; Jahan and Yumura, 2017), but similar events have also been described in cultured mammalian cells (Kanada M et al., 2005; Choudhary A et al., 2013; Gupta D K et al., 2018). However, mammalian cells rarely undergo cytofission, and whether it will occur after a failed cytokinesis or if the cell instead will become tetraploid depends on if the narrow ingression furrow is present or not. If a mitotic cell has formed and maintained the furrow, the connected daughter cells will generate tension on the connecting bridge, owing to their independent migration from each other, which may result in cytofission. In cases where the ingression furrow does not form or regress, the binucleated cell will migrate as one unit and not undergo cytofission (Gupta D K et al., 2019) (Figure 3).

In cells that fail in cytokinetic abscission because of insufficient integrin signals, the ingression furrow remains for long times *in vitro* (days), although the midbody disintegrates within a few hours (Gupta D K et al., 2019). The slow depolymerization of septin filaments at the intercellular bridge appears to be responsible for the furrow’s stability. As a result, under disturbed integrin signaling conditions, the ingression furrow’s longevity increases the possibility of cytofission, which could serve as a protection mechanism against the formation of tetraploid cells (Choudhary A et al., 2013; Gupta D K et al., 2018). However, it should be noted that cytofission has not been studied *in vivo* and that this alternative mechanism of cell division may be hampered at locations with neighboring cells (Kanada M et al., 2005).

### 3.5 Cytokinesis and cancer

A characteristic feature of cancer cells is their ability to complete cytokinesis without integrin adhesion, which may facilitate colony formation at foreign locations during metastatic spreading (Thullberg M et al., 2007; Pellinen T et al., 2008; Desgrosellier and Cheresh, 2010; Deng Z et al., 2021). Identification of the adhesion-independent cytokinesis mechanism(s) used by cancer cells is of interest, particularly since it may potentially lead to the identification of tumor-specific treatment targets. Studies of colony growth in soft agar or spheroid formation from single cells in suspension culture show that many proteins (mutated or overexpressed) can promote such adhesion-independent growth. However, in these assays, it is difficult to distinguish whether the implicated protein was acting directly on cytokinesis or had other indirect effects on cell division, for example, the synthesis of ECM (Salmenpera P et al., 2008), which could allow “pseudo-anchorage-independent” growth (Gupta and Johansson, 2013). Interestingly, several lines of more direct research on cytokinesis indicate that Ras plays an important role in the process. *Dictyostelium* cells cannot undergo normal cytokinesis after inactivating the Ras gene (Tuxworth R I et al., 1997), the slime mold orthologue of mammalian K-Ras, but they can perform cytofission. In human fibroblasts, oncogenic H-Ras (a close relative to K-Ras) was reported to promote anchorage-independent cytokinesis (Thullberg M et al., 2007). Later the detailed analysis showed that expression of the active Ras mutant specifically overcomes the abscission block in the detached cells by allowing the recruitment of Alix to the MB followed by Chm4B-mediated membrane fusion (Gupta D K et al., 2019).

On the other hand, strongly elevated levels of Ras activity can also impair the cytokinesis (Yang G et al., 2013; Duhamel S et al., 2016). Several studies have shown that this effect is mediated *via* the elevated protein levels of Aurora A (Yang G et al., 2013; Duhamel S et al., 2016; Umstead M et al., 2017; Chiu S-C et al., 2019). The present data indicate that Ras and Aurora A are essential regulators of cytokinetic abscission and that too low or too high activity of these proteins will impair the process.

## 4 Cytokinesis failure and their consequences

**FIGURE 4:**
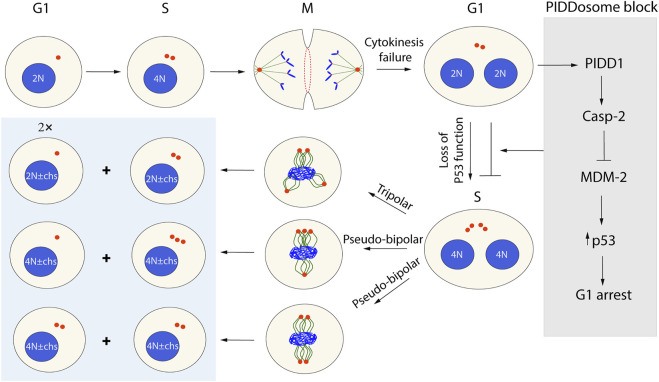
Possible outcomes of cytokinesis failure in the next cell cycle round. Cells that failed cytokinesis because of different abnormalities become tetraploid with supernumerary centrosomes (two mature centrosomes). In the G1 phase, the presence of more than one mature centrosome will activate PIDD1 and trigger PIDDosome-induced cell cycle arrest of normal cells. However, some viral infections and p53 mutations allow the cells to bypass the PIDDosome block (gray box) and proceed through the cell cycle. In the M phase, such cells may die or form a heterogenous progeny of tetraploids or aneuploids (blue box), depending on how the centrosomes will be arranged.

As described above, mitotic exit is a prerequisite for cytokinesis. In addition to insufficient integrin signaling, several other conditions can cause cytokinesis failure, including defects in the bipolar spindle formation caused by a variety of errors such as mitotic slippage, lagging chromosomes blocking the membrane fusion, and mutations (Ganem N J et al., 2009; Godinho S A et al., 2009). The most prominent and immediate consequence of cytokinesis failure is the formation of a tetraploid daughter cell with two centrosomes (Lacroix and Maddox, 2012). The tetraploid state is unstable and often develops into aneuploidy as a result of a continued cell cycle with more than two centrosomes, leading to the miss-segregation of chromosomes. Such aneuploid cells are often viable because the loss of genes is more tolerable in the tetraploid state containing the additional copies of the same gene that buffer the loss (Ganem N J et al., 2007; Lens and Medema, 2019). However, while aneuploidy is commonly found in cancer cells, most somatic cells have normal ploidy due to the induction of p53-dependent cell cycle arrest in the G1 phase following a cytokinesis failure incidence. Cell cycle arrest or apoptotic cell death in the G1 phase is primarily controlled by the tumor suppressor p53, which can be regulated by p53-induced protein with a death domain 1 (PIDD1) (Thompson and Compton, 2010; Fava et al., 2017; Burigotto and Fava, 2021). Upon activation, PIDD1 recruits both RAIDD and caspase-2 (CASP2) to form a multiprotein complex known as the PIDDosome. Recent studies have shown that the PIDDosome assembly is dependent on PIDD1 interaction with the centriole distal appendage protein ANKRD26 (Evans L T et al., 2021), which is only present in the fully mature mother centriole. ANKRD26 recruits PIDD1 when two (or more) mature centrosomes are present in a cell after cytokinesis failure, possibly due to the merging of the centrosomes (Fava et al., 2017; Evans et al., 2021). The assembly of the PIDDosome complex promotes the autocatalytic, proximity-induced activation of CASP2. Activated CASP2 stabilizes p53 *via* cleavage of MDM2 and causes the subsequent upregulation of the cell cycle inhibitor p21 by p53 (Thompson and Compton, 2010; Fava L L et al., 2017; Burigotto and Fava, 2021; Evans L T et al., 2021). Cells with inactivated p53, similar to many cancer cells (Davoli and De Lange, 2011), bypass the PIDDosome block and enter the subsequent mitosis with more than two centrosomes. To avoid multipolar spindle formation, the cells must cluster the supernumerary centrosomes into two polar groups forming a pseudo-bipolar spindle. However, since pseudo-bipolar spindles, as well as tri- or multi-polar spindles, increase the frequency of faulty chromosome segregation, aneuploid cells can be formed (Figure 4).

## 5 Future perspectives

The understanding that integrin-mediated adhesion is required for cytokinetic abscission in normal cells and for separating the two centrosomes to opposite sides of the nucleus to form the mitotic spindle raises several questions. The most immediate question regards the involvement of FAK in the spatio-temporal regulation of PLK1 activity. PLK1 regulates the centrosome separation, and the upstream links between the activation of PLK1 and FAK need to be identified. The contribution of FAK to MB maturation may also concern the regulation of PLK1 since the blocked recruitment of Alix and TSG101 to Cep55 correlates with a strongly enhanced degradation of PLK1 during cytokinesis in cells with reduced FAK activity.

Another critical question is how Ras and Aurora A can promote or prevent cytokinesis dependent on their activity levels and, in particular, how oncogenic Ras mutants can induce mitotic abscission in non-adherent cells. Although more indirectly connected to integrin signals, it would be interesting to clarify whether the MB-independent cell division by cytofission observed *in vitro* also is a phenomenon occurring *in vivo*. The cytofission *in vitro* depends on the slow septin depolymerization at the intercellular bridge, a mechanism that needs to be understood and waiting for clarification. Additionally, many questions can be addressed regarding the regulation of centrosome functions since, as indicated in this review, in case of failed cytokinesis due to insufficient adhesion signals or other causes, the centrosomes will have central roles in the fate of the cell.
